# Placental trophoblast debris mediated feto-maternal signalling via small RNA delivery: implications for preeclampsia

**DOI:** 10.1038/s41598-017-14180-8

**Published:** 2017-10-31

**Authors:** Jia Wei, Cherie Blenkiron, Peter Tsai, Joanna L. James, Qi Chen, Peter R. Stone, Lawrence W. Chamley

**Affiliations:** 1 Department of Obstetrics and Gynaecology, The University of Auckland, Auckland, New Zealand; 2 Department of Surgery, The University of Auckland, Auckland, New Zealand; 3 Department of Molecular Medicine and Pathology, The University of Auckland, Auckland, New Zealand; 4 Department of Obstetrics and Gynaecology, Tongji Hospital affiliated to Tongji Medical College, Huazhong University of Science and Technology, Wuhan, 430030 People’s Republic of China

## Abstract

To profile the small RNA cargo carried by trophoblast debris derived from the placenta during normal and preeclamptic pregnancies and to determine whether trophoblast debris can deliver its small RNAs to endothelial cells with functional consequences. We confirmed that trophoblast debris can deliver its small RNAs contents to recipient endothelial cells during the co-culture. Next generation sequencing was employed to profile the small RNA contents in both normotensive and preeclamptic trophoblast debris. We identified 1278 mature miRNAs and 2646 non-miRNA small RNA fragments contained. Differential expression analysis identified 16 miRNAs (including miR-145), 5 tRNA fragments from 3 different tRNAs, 13 snRNA fragments and 85 rRNA fragments that were present in different levels between preeclamptic and normotensive trophoblast debris. We loaded a miR-145 mimic into normotensive trophoblast debris via transfection of placental explants from which the debris was derived and found the miR-145 loaded debris induced transcriptomic changes in endothelial cells similar to those induced by preeclamptic trophoblast debris. Trophoblast debris deported into maternal circulation can deliver its small RNA contents to maternal cells thereby contributing to feto-maternal communication. Small RNAs that are dysregulated in preeclamptic trophoblast debris might contribute to the endothelial cell activation which is a hallmark of preeclampsia.

## Introduction

The entire human placenta is covered by a single multinucleated cell, the syncytiotrophoblast, which is a terminally differentiated epithelium formed by the fusion of the cytotrophoblasts. Most if not all cells produce extracellular micro-vesicles (100–1000 nm) and nano-vesicles (70–120 nm including exosomes), that function in intracellular communication. Like other cells, the syncytiotrophoblast also produces micro- and nano-vesicles, but due to the unique multinucleated nature of this cell, the placental syncytiotrophoblast also produces material traditionally referred to as trophoblast debris althought growing evidence suggests the term debris is a misnomer^1^. Trophoblast debris consists of very large syncytiotrophoblast derived vesicles called syncytial nuclear aggregates (SNA) which vary in size from 20–200 μm and contain between 2 and several hundred nuclei, mononuclear cytotrophoblasts and anucleate trophoblast “ghosts”^1^. The syncytiotrophoblast continuously extrudes large quantities of each of these types of extracellular vesicles into the maternal blood which deports them from the placental site throughout pregnancy. For decades, the deportation of placental extracellular vesicles was proposed to be nothing more than a mechanism by which the human placenta eliminated aged/non-functional contents of the syncytiotrophoblast. However, it is becoming increasingly clear that placenta-derived extracellular vesicles/trophoblast debris are an important mechanism by which the fetus communicates with its mother during both normal and complicated pregnancies such as preeclampsia. Preeclampsia is a human-pregnancy-specific hypertensive disorder which is triggered by a factor(s) released from the placenta^1–4^.

Placental extracellular vesicles carry a large array of proteins, nucleic acids and lipids that could mediate feto-maternal communication and recently we have shown that trophoblast debris can induce the expression of placenta-specific genes in endothelial cells although the mechanism of this induction was not clear^1^. Others have shown that viral resistance can be induced in otherwise susceptible cells by transfer of miRNAs from trophoblasts to the susceptible cells via exosomes^5^.

MicroRNAs are one type of small non-coding RNA (ncRNAs). Small ncRNAs can be classified into microRNA (miRNAs), small nucleolar RNA (snoRNA), small nuclear ribonucleic acid (snRNA), piwi-interacting RNA (piRNA) and transfer RNA (tRNA), with miRNA being the most studied subclass. MicroRNAs can suppress gene expression by either, degradation of target mRNAs or, repression of translation through sequence complementary between the RNA-induced silencing complex (RISC) and the target mRNA^6–10^. Approximately 100 miRNAs are produced only in the placenta^11^ and they are mostly localized within three miRNA clusters: chromosome 14 miRNA cluster (C14MC), chromosome 19 miRNA cluster (C19MC) and miR-371-3 cluster^11–13^. The function of these miRNA clusters is not fully understood, but evidence suggests that they are involved in human embryonic stem cell development and trophoblast proliferation, invasion and differentiation^14–17^.

A number of studies have identified that miRNAs are differentially expressed in either the placenta or maternal plasma between preeclamptic and healthy pregnancies^18–21^. However, none of these studies have examined whether trophoblast debris contains miRNAs or whether there are different levels of specific miRNAs in the trophoblast debris from preeclamptic or normotensive placentae. Therefore, the aims of this study were to; **1**) confirm that, like the smaller extracellular vesicles, trophoblast debris does contain small RNAs; **2**) identify small RNAs that are present at different levels in the trophoblast debris from preeclamptic and normotensive placentae; **3**) confirm that miRNAs that are present at significantly different levels between trophoblast debris from preeclamptic and normotensive placentae can be transferred to (maternal) endothelial cells and affect the function of those cells.

## Results

### Delivery of small RNA into endothelial cells by trophoblast debris

In order to determine whether trophoblast debris was capable of delivering small RNAs to endothelial cells, placental explants were transfected with a cy3-labelled scrambled siRNA and the trophoblast debris was collected after culture for 21 hours.

**Figure 1:**
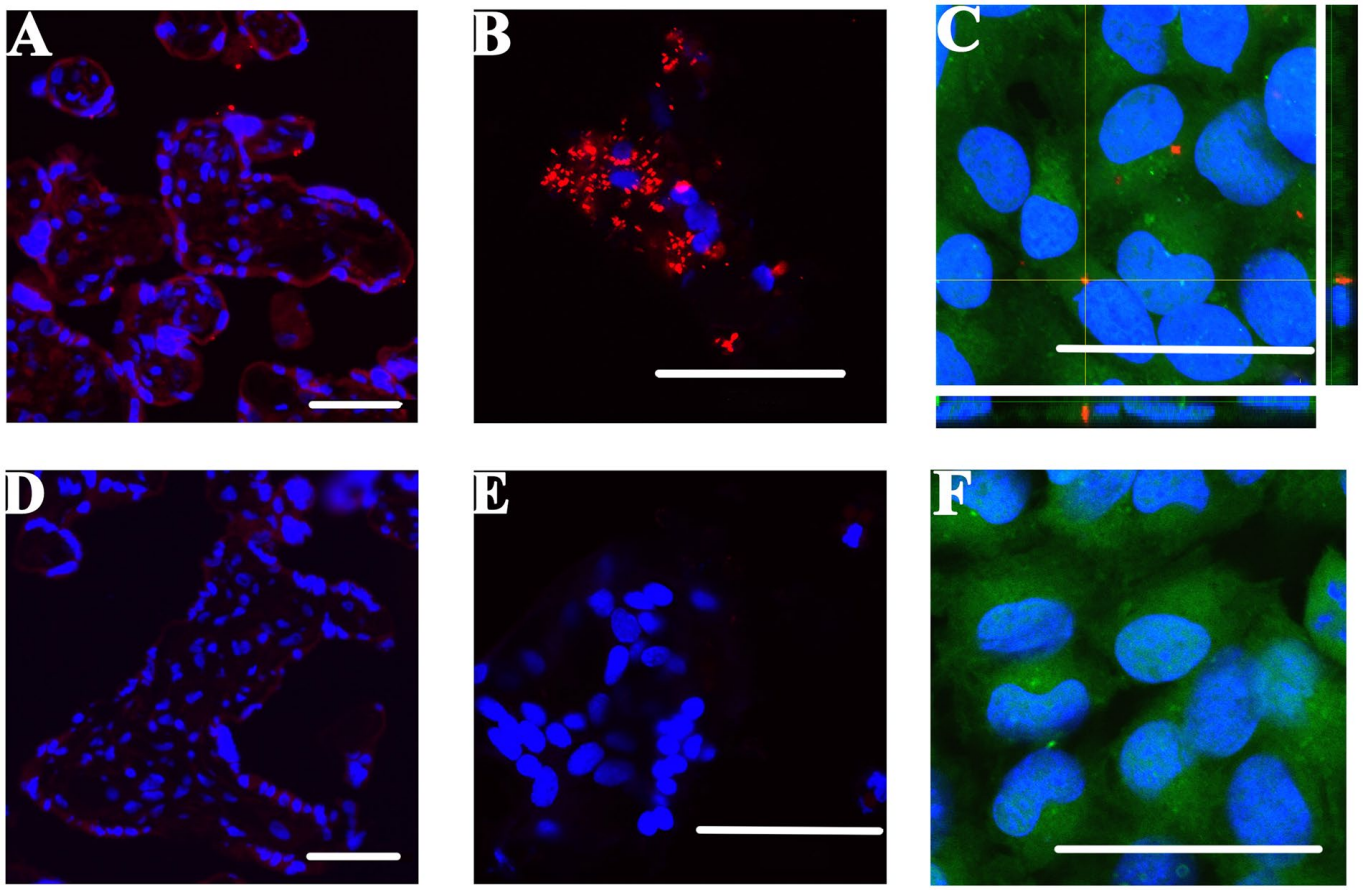
Visualizing delivery of fluorescent siRNA to placental explants, trophoblast debris and transfer to endothelial cells by trophoblast debris. Normotensive term placental explants (n = 3) were cultured with (**A**,**B**) or without (bottom panel: D,E) the presence of 25 pmol of cy3 labelled siRNA (red) and lipofectamine mixture for 17 hours. Five µm sections were cut from the explants (**A**,**D**) and smears of trophoblast debris from the explants (**B**,**E**) were co-stained with DAPI nuclear marker (blue) and visualised by confocal microscopy. HMEC-1 cells were exposed to trophoblast debris from cy3-siRNA containing (**C**, red) or untreated trophoblast debris (**F**) for 21 hours before co-staining with Cell Tracker Green (CMFDA, green) and DAPI (blue). The main panel of image C shows the projection image of the z-stack. The bottom panel shows the Z-projection of X-Z axis, while the panel on the right shows the Z-projection of Y-Z axis. Scale bars represent 50 μm in all images.

Confocal microscopy imaging showed that the cy3-siRNA was predominantly localized to the syncytiotrophoblast of the placental explants (Fig. 1A). Cy3-siRNA was also present in the trophoblast debris extruded from these cy3-siRNA transfected placental explants, with most of the cy3-siRNA being localized to the cytoplasm of the trophoblast debris (Fig. 1B).

The trophoblast debris containing the cy3-labelled siRNA was then exposed to HMEC-1 endothelial cells and confocal microscopy showed that cy3-siRNA was able to be delivered to the endothelial cells via trophoblast debris in the absence of any transfection reagents (Fig. 1C). The siRNA was localized in peri-nuclear clusters in the endothelial cells.

### Identification of the small RNA contents of preeclamptic and normotensive trophoblast debris

RNA electrophoresis using an Agilent 4200 Tape Station system showed that the length of the majority of the RNA contained in the preeclamptic and normotensive trophoblast debris was below 200 nucleotides. Distinct ribosomal RNA peaks were not readily identifiable, resulting in low RIN (RNA Integrity Number) values for these samples (1.88 ± 0.59, n = 4, Supplementary Figure 1).

The small RNAs present in trophoblast debris from four preeclamptic and four normotensive placentae was assessed and quantified by small RNA deep sequencing. Approximately 20 million total reads were generated from each sample library.

**Figure 2:**
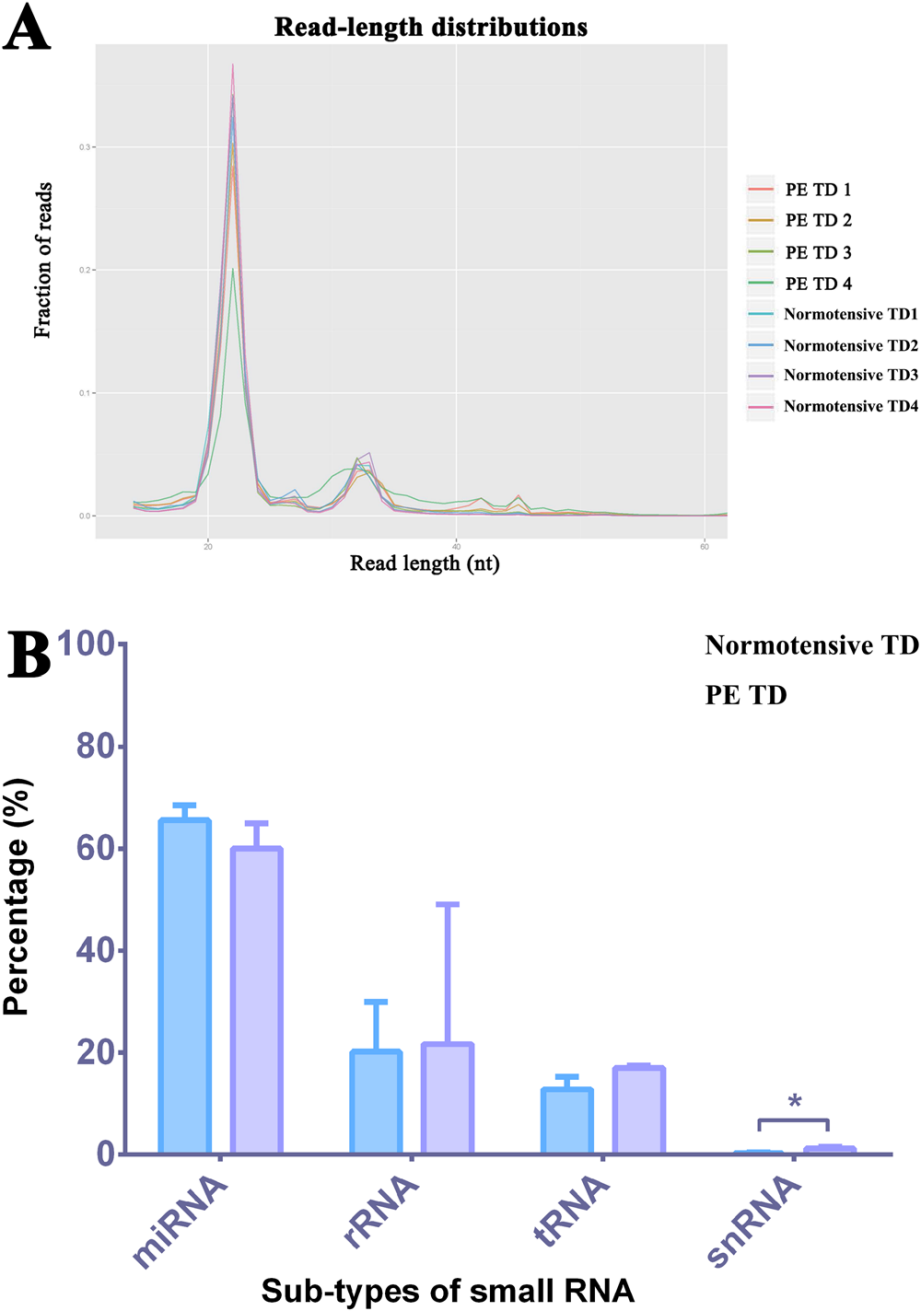
Read length distribution and the composition of endogenous small RNAs contained within preeclamptic and normotensive trophoblast debris. (**A**) The plot indicates the read-length distribution generated from the small RNA sequencing of the trophoblast debris isolated from four preeclamptic and four control placentae. The x-axis indicates the length of small RNA reads, while the y-axis indicates the percentage of the small RNA reads with specific lengths for the total reads. PE = preeclamptic, TD = trophoblast debris. (**B**) Total sequence reads were annotated to small RNA databases (miRNA, tRNA. rRNA, and snRNA) for the human genome, version 38. The bar graphs show the percentage of different types of small RNAs in the total small RNA reads and contrasts the percentages in preeclamptic and normotensive trophoblast debris.

The read-length distribution plot (Fig. 2A) showed that the majority of the sequences were 20–24 nucleotides in each library, which is the average size of mature miRNA. A small peak was also seen at 32 nucleotides, which indicates the presence of other small RNA fragments. Sequences were then annotated to human genome, version 38, to identify miRNA and fragments from other small RNAs including tRNA, snoRNA and rRNA, using the iSRAP small RNA pipeline. Two thousand six hundred and forty-six non-miRNA small RNA fragments and 1278 mature miRNAs were identified across all of the samples. The small RNA sequence composition showed that the majority of these sequences were miRNAs (60–65%), while fragments from rRNA were the second largest small RNA population in trophoblast debris (20.2–21.7%) and tRNA fragments accounted for 12.8 to 17% of the total small RNA population. When comparing the small RNA composition between preeclamptic and control trophoblast debris, snRNA fragments were significantly more abundant in preeclamptic trophoblast debris compared to control (1.27% vs. 0.28%, p-value = 0.027, n = 4) (Fig. 2B). No fragments from snoRNA were identified.

### Differences in the levels of individual small RNAs between preeclamptic and normotensive trophoblast debris

To ensure robust analysis, we used three normalization methods based on three different packages: DESEq. 2, EdgeR and Voom provided by iSRAP pipeline (adjusted p-value or false discovery rate <0.05 and log2 fold change > ±1) to identify the small RNAs present at different levels between the normotensive and preeclamptic trophoblast debris. Only those small RNAs that were identified using all three methods were considered to be present at different levels. Thus, 103 non-miRNA small RNAs fragments were present in different levels in preeclamptic and control trophoblast debris; 85 of which were rRNA fragments (Supplementary Table 3), 5 of which were tRNA fragments from 3 different tRNAs and 13 of which were snRNA fragments (Supplementary Table 4).

Similarly, 16 miRNAs were consistently identified as being present at different levels between preeclamptic and normotensive trophoblast debris regardless of the normalization method used. Among these, 15 miRNAs were present at significantly higher levels and 1 miRNA (has-miR-4443) was present at significantly lower levels in preeclamptic compared to normotensive trophoblast debris. Of note, the expression of miR-1247-5p and miR-526b-5p from the placenta-specific, C14MC and C19MC, clusters respectively, were present at higher levels in preeclamptic trophoblast debris (Table 1).

**Table 1:** MicroRNAs that were present at significantly different levels between preeclamptic and control term trophoblast debris.

miRNA	DESeq. 2		EdgeR		Voom	
	Log2 fold change	Adjusted P-value	Log2 fold change	FDR	Log2 fold change	Adjusted P-value
hsa-miR-615-3p *	2.301	0.013	3.782	0.011	3.107	0.026
hsa-miR-455-5p *	2.024	0.015	2.703	0.013	2.986	0.031
hsa-miR-3178	2.042	0.047	5.715	0.014	2.917	0.031
hsa-miR-27a-5p	1.826	0.048	2.481	0.046	2.855	0.032
hsa-miR-199b-5p	2.316	<0.001	2.664	0.002	2.433	0.022
**hsa-miR-1247-5p** *	2.193	<0.001	2.513	0.002	2.309	0.024
hsa-miR-145-5p *	2.066	<0.001	2.293	0.002	2.122	0.022
hsa-miR-143-3p	1.731	0.005	2.021	0.006	2.075	0.024
hsa-miR-199a-5p	1.918	<0.001	2.151	0.004	2.041	0.026
hsa-miR-199a-3p	1.639	0.005	1.879	0.006	1.883	0.024
hsa-miR-199a-5p	1.842	0.001	2.082	0.006	1.872	0.041
hsa-miR-193b-5p	1.521	0.008	1.723	0.013	1.851	0.041
*hsa-miR-526b-5p* *	1.557	0.003	1.76	0.002	1.825	0.022
hsa-miR-199b-3p	1.538	0.008	1.76	0.007	1.789	0.026
hsa-miR-199a-3p	1.515	0.012	1.744	0.011	1.775	0.031
hsa-miR-126-3p	1.637	<0.001	1.795	0.002	1.682	0.024
hsa-miR-30a-3p	1.391	0.008	1.542	0.01	1.551	0.041
hsa-miR-4443	−1.771	0.001	−1.915	0.004	−1.705	0.032

Note: Micro RNAs encoded from the C14MC are labelled **bold**, and miRNAs belonging to C19MC are labelled *Italic* in column one. FDR = false discovery rate. MicroRNAs followed with *were the ones validated by qRT-PCR.

### Validation of the differences in miRNAs between preeclamptic and normotensive trophoblast debris using qRT-PCR

**Figure 3:**
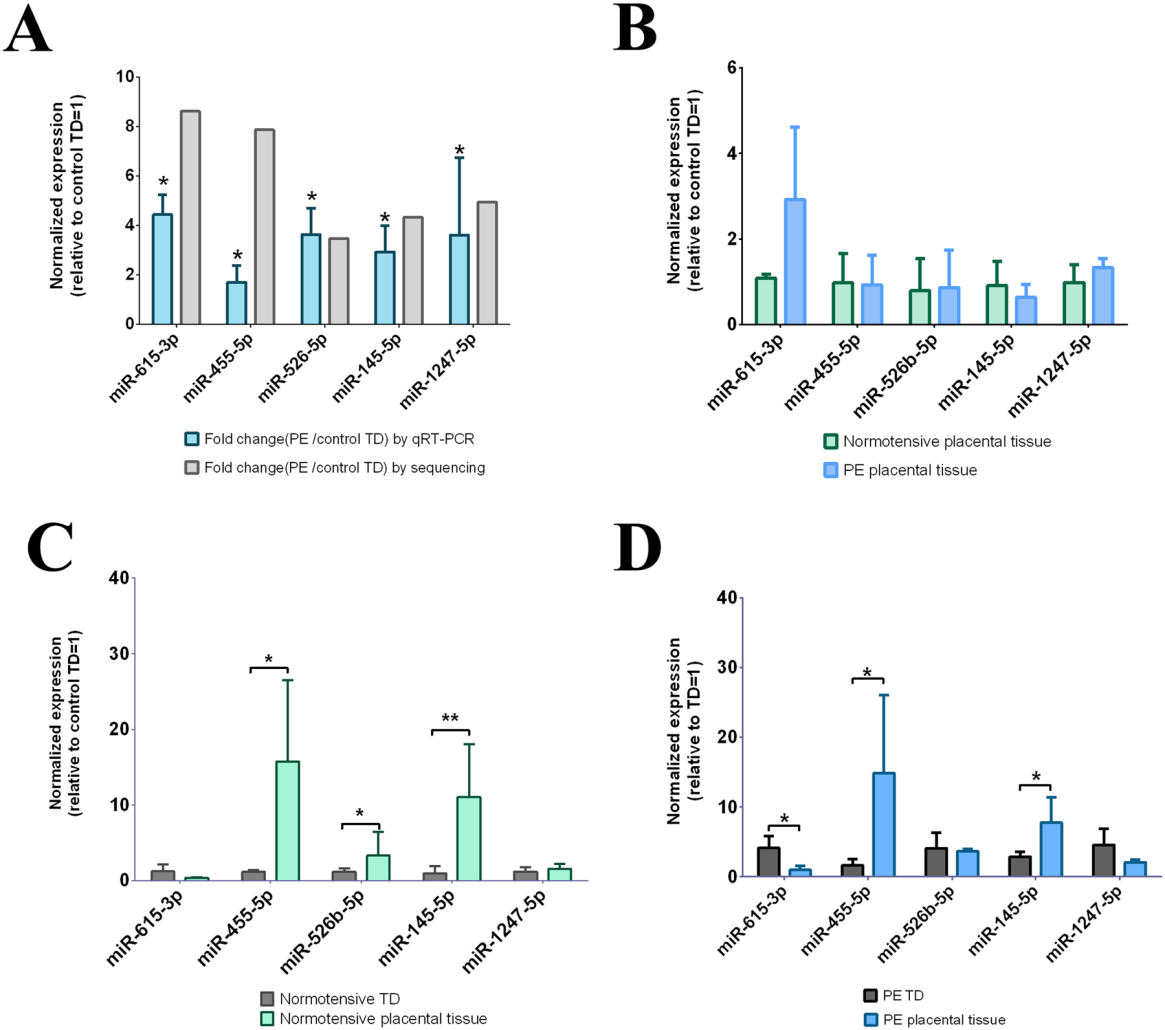
Quantification of the miRNAs that were present at different levels in preeclamptic and normotensive trophoblast debris, as well as the placental explants from which they were derived. To validate the small RNA sequencing results, the levels of 5 miRNAs in both preeclamptic and control normotensive trophoblast debris, as well as the placental explants from which they were derived were quantified by qRT-PCR. The expression of miRNA was normalized to the geometric mean of three reference miRNAs (miR-532-5p; miR-92b-3p; miR-29c-5p). Data is presented as median ± interquartile range. *indicates P-value from Mann Whitney test < 0.05; **indicates P_Mann_ < 0.01. PE = preeclampsia; TD = Trophoblast debris. (**A**) Comparison of the levels of 5 miRNAs between preeclamptic (n = 7) and normotensive trophoblast debris (n = 4). The bar graphs represent the fold change for the expression of miRNA in preeclamptic (PE) trophoblast debris (TD) compared to control TD by either qRT-PCR or small RNA sequencing. (**B**) Comparing the levels of 5 miRNAs between preeclamptic (n = 5) and normotensive placental explants (n = 4); (**C** and **D**) Comparison of the levels of 5 miRNAs between trophoblast debris and the matched donor placental explants form during normotensive (**C**, n = 4) and preeclamptic pregnancies (**D**, n = 5).

In order to validate the differences seen in the deep sequencing analysis, the levels of 5 miRNAs (miR-615-3p; miR-455-5p; miR-526b-5p; miR-145-5p; miR-1247-5p) identified as being present at higher levels in preeclamptic than normotensive trophoblast debris were quantified by qRT-PCR. Quantitative RT-PCR showed that preeclamptic trophoblast debris contained significantly higher levels of miR-615-3p (p < 0.015), miR-455-5p (p < 0.025), miR-526b-5p (p < 0.035), miR-145-5p (p < 0.025) and miR-1247-5p (p < 0.03) compared to control trophoblast debris (Fig. 3A).

### Comparison of the levels of miRNAs between trophoblast debris and the placental tissue from which it was derived

In order to compare the levels of the same five miRNA between preeclamptic and normotensive placental tissue from which the trophoblast debris was derived, the levels of these miRNAs were also quantitated in the donor placental tissue. However, none of the miRNA candidates were present at significantly different levels in preeclamptic compared to normotensive placental explants (Fig. 3B).

Comparison of the levels of the five miRNAs between the preeclamptic placental explants and the debris derived from them, showed that the levels of miR-455-5p and miR-145-5p were significantly lower in the trophoblast debris than the placenta from which it was derived, while the levels of miR-615-3p were significantly higher in preeclamptic trophoblast debris than in the placental tissue from which it was derived (Fig. 3D and Table 2).

**Table 2:** The expression of miRNAs in trophoblast debris and the placental explants from which the debris was derived in preeclamptic and normal pregnancies.

miRNA	Trophoblast debris	Matched donor placental explants	p-value	Numbers
**Normal pregnancies**				
miR-455-5p↓	1.04 ± 0.41	16.05 ± 10.95	<0.05	4
miR-526b-5p↓	1.11 ± 0.52	4.19 ± 2.18	<0.05	4
miR-145-5p↓	1.15 ± 0.75	12.17 ± 5.67	<0.01	4
**Preeclamptic pregnancies**				
miR-455-5p↓	1.91 ± 0.59	17.08 ± 10.72	0.05	5
miR-145-5p↓	2.73 ± 1.06	7.92 ± 3.49	0.05	5
miR-615-3p↑	3.93 ± 2.02	0.91 ± 0.76	0.05	5

Note: The arrow next to each miRNA indicates the directional change of the level of this miRNA in trophoblast debris compared to the placental explant from which the debris was derived.

Quantitative RT-PCR analysis showed that the levels of miR-455-5p, miR-526b-5p and miR-145-5p were significantly lower in normotensive trophoblast debris than in the placental explant from which the debris was derived (Fig. 3C and Table 2).

### Engineering normotensive trophoblast debris into preeclamptic–like trophoblast debris by loading with miRNA mimics

In order to interrogate the potential effect of the increased level of miRNAs in preeclamptic trophoblast debris, three miRNAs (miR-526b-5p and miR-1247-5p belonging to the C19MC and C14MC respectively and miR-145-5p) that were shown to be present at increased levels in preeclamptic debris were used to engineer preeclamptic-like trophoblast debris.

To do this, normotensive third trimester placental explants were transfected with synthetic miRNA mimics of these three miRNAs and the trophoblast debris derived from the placental explants was collected and divided into two portions: one of which was used for quantification of the delivery efficiency, and the other was added to cultures of endothelial cells.

**Figure 4:**
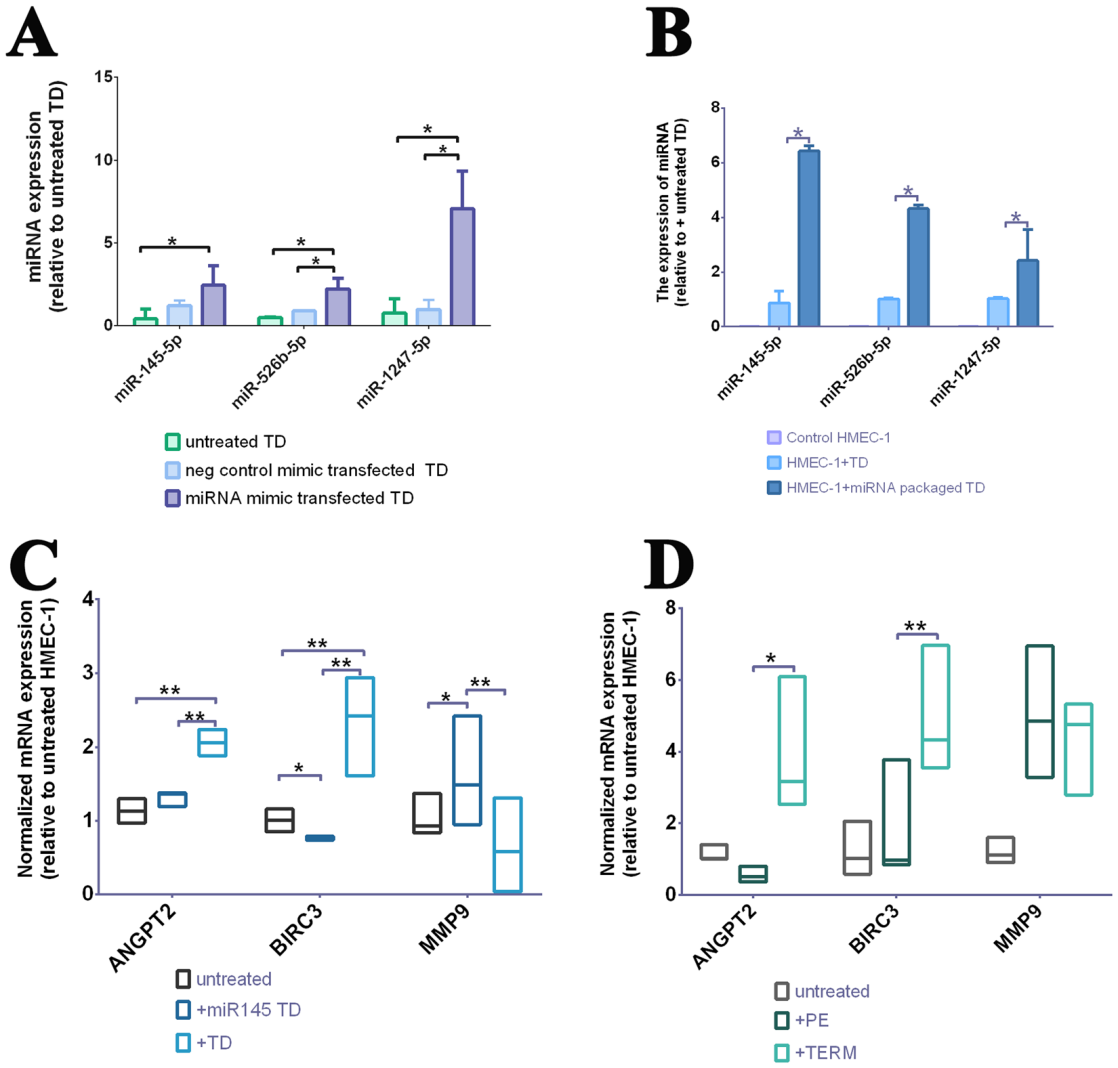
Quantification of the level of delivered synthetic miRNA mimics in trophoblast debris and HMEC-1 cells, as well as the expression of predicted targets of miR-145 in HMEC-1 cells after exposure to miR-145-mimic-containing or preeclamptic trophoblast debris. The levels of three synthetic miRNAs that were present in trophoblast debris after transfection of placental explants (**A**) and level of the three mimics in endothelial cells that had been exposed to the mimic–containing trophoblast debris (**B**) were quantified by qRT-PCR. The expression of miRNA was normalized to the geometric mean of three reference miRNAs (miR-532-5p; miR-92b-3p; miR-29c-5p). Data is presented as median ± interquartile range. *indicated P-value from Mann Whitney test <0.05. TD = Trophoblast debris. Expression in endothelial cells of three predicted target genes of miR-145 after exposure to either miR-145 mimic-containing (**C**) or preeclamptic trophoblast debris (**D**) were quantified by qRT-PCR. The expression of mRNA was normalized to the geometric mean of two housekeeping genes (*ACTB, 18 S*). Data is presented as median ± interquartile range. *indicated P-value from Mann Whitney test <0.05; **indicates P_Mann_ < 0.01. TD = Trophoblast debris.

Quantitative RT-PCR confirmed that engineered trophoblast debris derived from miRNA mimic-transfected placental explants contained significantly increased levels of miR-145-5p (2.44 *vs*. 0.41, n = 3, p-value < 0.01), miR-526b-5p (2.23 *vs*. 0.50, n = 3, p-value < 0.01) and miR-1247-5p (7.06 *vs*. 0.76, n = 3, p-value < 0.01) than trophoblast debris from untreated placental explants (Fig. 4A). These increased levels of the mimics were similar to the fold changes seen in the levels of the corresponding miRNAs in our deep-sequencing data. As expected, there were basal (endogenous) levels of these miRNAs in the trophoblast debris from untreated explants but there was no significant difference in the levels of the three miRNAs between the two controls (Fig. 4A).

Endothelial cells exposed to miRNA mimic-containing trophoblast debris also contained significantly higher levels of miR-145-5p (p-value < 0.001), miR-526b-5p (p-value < 0.001) and miR-1247-5p (p-value < 0.001) compared to endothelial cells treated with trophoblast debris from untreated explants (Fig. 4B).

We next investigated the functional consequences of delivering excess miR-145 into endothelial cells by quantifying the levels of transcripts for three target of miRNA 145: *ANGPT2*, *BIRC3* and *MMP9* in HMEC-1 cells that had been exposed to miRNA145-mimic-containing normal trophoblast debris or preeclamptic debris. Endothelial cells exposed to miR-145 mimic-containing trophoblast debris expressed significantly lower levels of *ANGPT2* (p-value < 0.001) and *BIRC3* (p-value < 0.005) mRNA relative to HMEC-1 cells exposed to normal trophoblast debris. Conversely, the expression of *MMP9* was significantly higher in endothelial cells treated with miR-145 mimic-containing trophoblast debris compared to endothelial cells treated with normal trophoblast debris (p-value < 0.005, Fig. 4C).

Similar expression changes in miRNA145-targets were also seen when endothelial cells were treated with preeclamptic trophoblast debris. They expressed significantly lower levels of *ANGPT2* (p-value < 0.01) and *BIRC3* (p-value < 0.005) compared to endothelial cells exposed to normotensive trophoblast debris, while the expression of MMP9 was not different between HMEC-1 cells exposed to preeclamptic or normotensive trophoblast debris (p-value < 0.05, Fig. 4D).

## Discussion

While the biogenesis of trophoblast debris is not well understood, for many decades it was thought that this material was worn out or damaged regions of the syncytiotrophoblast and thus production of trophoblast debris was simply a mechanism by which the syncytiotrophoblast rid itself of aged or non-functional regions. Now it is increasingly accepted that extracellular vesicles derived from the human placenta, including trophoblast debris, play an important role in feto-maternal signalling. Their reported involvement ranges from regulating maternal endothelial cell migration to immune cell activation and transfer of resistance to viral infection^2,22,23^. While the majority of research has focused on smaller microvesicles, nanovesicles, and exosomes derived from the placenta, we have recently shown that the much larger vesicles, trophoblast debris, could also contribute to the normal maternal cardiovascular adaptation to pregnancy following the phagocytosis of these vesicles by endothelial cells^1^.While others have shown that exosomes can transfer their miRNA cargos to recipient cells the mechanism by which exosomes are taken up by target cells are different to the phagocytic mechanism that appears to be important in the uptake of trophoblast debris by endothelial cells and macrophages^24,25^. Here, using a model system employing fluorescently-labelled siRNA, we have shown that trophoblast debris can deliver small RNAs into endothelial cells (Fig. 1C, Supplementary Figure 2). The mechanism of this uptake of small RNAs carried by trophoblast debris could be by phagocytosis of the debris, or alternatively, via uptake nanovesicles/microvesicles released from trophoblast debris. Supporting the latter concept, it has recently been published that many syncytial nuclear aggregates (the largest, multinucleated vesicles in trophoblast debris) produce blebs on their surface that may well be shed as microparticles^26,27^. It has previously been reported that the syncytiotrophoblast can be transfected with siRNA either with or without the use of a transfection reagent^28^. We confirmed that result here and extended it to show that siRNA once transfected into the syncytiotrophoblast is then packaged into the trophoblast debris that is extruded from the transfected syncytiotrophoblast. It is also possible that the siRNA was transfected directly into the trophoblast debris after the debris was extruded from the syncytiotrophoblast. Previous work using this explant model to generate trophoblast debris demonstrated that approximately 50% of the debris collected after 20 hours of explant culture was able to metabolise the green fluorescent stain CMFDA suggesting that at least some trophoblast debris retains metabolic activity and as such, may have the capacity to be directly transfected^26^. Alternatively, the siRNA could enter the debris by both of these routes and that the debris appeared to have stronger staining for the siRNA than the syncytiotrophoblast indicating that both routes might be involved (Fig. 1A,B).

The syncytial nuclear aggregates present in trophoblast debris are very large structures with on average approximately 60 nuclei and an equal volume of nuclei and cytoplasm^27^. Therefore, they each have the potential to carry vast quantities of RNA. Our analysis of the RNA extracted from trophoblast debris demonstrated that there was relatively little intact (large) mRNA (Supplementary Figure 1). Never the less, deep sequencing confirmed abundant small RNAs were present in the trophoblast debris. In total, we identified some 3924 small RNAs in trophoblast debris, including miRNA and fragments of rRNA, tRNA, and snRNA. That the major species of small RNA present in trophoblast debris was miRNA suggests that trophoblast debris may play an important role in distributing this regulatory RNA from the placenta to the mother.

Using the iSRAP small RNA analysis pipeline, we were able to determine that fragments from rRNA, tRNA and snRNA, as well as miRNAs were present at different levels between preeclamptic and normotensive trophoblast debris. Ribosomal RNA and tRNA are known to be involved in protein translation, while snRNAs are demonstrated to play an important role in mRNA splicing^29^. Therefore, these small RNAs are all associated with transcriptional regulation, and possibly involved in placental/ fetal development. For a long time, the fragments from these small RNAs were thought to be a waste product of RNA degradation. However, accumulating evidence indicates that tRNA fragments are not random by-products of tRNA degradation or biogenesis, but rather that they have unique expression patterns and biological roles^30^ including translation control, RNA silencing, cell proliferation and regulation of apoptosis^31–34^. The biological role of these small RNA fragments in feto-maternal communication is not at all clear, nor has their contribution to the pathogenesis of preeclampsia been investigated and their potential role in pregnancy and/or preeclampsia requires further investigation.

Among the miRNAs that were present at different levels between preeclamptic and normotensive trophoblast debris, there were several that have previously been reported to be differentially expressed in the placentae of normotensive and preeclamptic pregnancies such as, the up-regulation of, miR-193b, miR-526b in preeclamptic trophoblast debris as seen in preeclamptic placentae^35,36^. However, the majority of the miRNAs that we found at different levels in preeclamptic trophoblast debris were not previously reported to be differentially expressed in either preeclamptic placental tissue or maternal plasma. These differences could be due to various reasons, including patients’ ethnicities, experimental differences (microarray vs. small RNA sequencing) and analysis methods applied. However, another very important difference is that we examined the miRNAs in trophoblast debris while these other studies examined placental tissue or plasma samples. Others have demonstrated selective packaging of small RNAs in microvesicles and exosomes, compared to the cells from which they are derived^37–39^. The mechanisms of small RNA sorting into extracellular vesicles are not fully understood. However, one recent study suggested that post-transcriptional modifications, notably 3′ end adenylation and uridylation, exert opposing effects that may contribute to direct non-coding RNA sorting into exosomes^39^. Here, qRT-PCR analysis comparing individual miRNAs in the trophoblast debris and the placental explants from which the debris was derived demonstrated that, the miRNA contents of debris was different to the content of miRNAs in the explants. While miR-615 was selectively enriched in the preeclamptic trophoblast debris compared to the matched placental tissue, some miRNAs were of low abundance in the debris (miR-497, miR-455 and miR-145) compared to the explant. That not all miRNAs were enriched in the trophoblast debris suggests that there may be selective packaging into the trophoblast debris (rather than simple protection of the miRNAs in the debris). The physiological function of selective small RNA packing into placental extracellular vesicles is not yet known. However, since there were substantial differences in the miRNA contents of preeclamptic and normotensive trophoblast debris it is tempting to speculate that selective packaging of small RNAs, especially miRNAs, could contribute to failure of the normal cardiovascular adaptation to pregnancy that is characteristic of preeclampsia.

Here for the first time, we have presented the contrasting profiles of miRNA carried by placental trophoblast debris during both normal and preeclamptic pregnancies. Our finding that the miR-143/145 cluster was present at higher levels in preeclamptic trophoblast debris than the control debris is consistent with the role of the cluster in regulating vascular homeostasis and blood pressure, as demonstrated by the observation that genetic deletion of this cluster significantly reduced blood pressure and vascular tone^40,41^. Conversely, up-regulation of miR-143/145 was found in atherosclerotic plaques from hypertensive patients and smooth muscle cells in pulmonary arterial hypertension^42,43^ and miRNA-143-3p enriched exosomes were able to change the behaviour of endothelial cells *in vitro*. These studies indicate that the elevated level of miR-143/miR-145 cluster contained within preeclamptic trophoblast debris might contribute to the high blood pressure through interaction with maternal endothelium. Given that of the miRNAs that we found to be present in different amounts between preeclamptic and normotensive trophoblast debris miR-145 has been well-studied and that it has been shown to have effects that are relevant to vascular function and preeclampsia we investigated the consequences of this increasing the amount of this miR in trophoblast debris using mimics transfected into the syncytiotrophoblast/trophoblast debris. The hypothesis that the miR143/145 cluster might be important in the pathogenesis of preeclampsia was reinforced here by showing that, artificially engineered miR-145 mimic-containing normotensive trophoblast debris was able to induce similar transcriptomic changes in the endothelial cells to those induced by preeclamptic trophoblast debris (Fig. 4C,D). While the expression of the miR-145-targets, *ANGPT2* and *BIRC3*, in endothelial cells was inhibited by the delivery of miR-145 mimic via trophoblast debris was expected^44–46^, the up-regulation of *MMP9* induced by the miR-145 mimic was unexpected. However, this result was consistent with the effect of preeclamptic trophoblast debris on endothelial cells confirming the biological relevance of our experiment. Considering that one miRNA can negatively regulate the expression of hundreds of genes^47^, there might be some positive effect of over expressing this single miRNA (miR-145) that was countered by the complex mixture of miRNAs and other signalling molecules contained within preeclamptic debris.

In summary, trophoblast debris, which is deported in large quantities into the maternal circulation every day during pregnancy, carries a large range of small RNAs especially miRNAs. This cargo of miRNAs is significantly different between the trophoblast debris from preeclamptic and normotensive pregnancies. Using a model system *in vitro*, we have demonstrated that trophoblast debris can deliver its small RNA cargo to maternal cells where those RNAs can regulate target gene expression in endothelial cells. If this *in vitro* phenomenon also occurs *in vivo*, miRNA delivered to maternal cells by trophoblast debris would be a mechanism by which the fetus could regulate distant maternal physiological functions and dysregulation of this system, by changes in the miRNA cargo of trophoblast debris, could contribute to the maladaptation to pregnancy that results in preeclampsia.

## Material and Methods

Informed consent involving human tissue used for research have been obtained from all donators. All experiments involving human tissue were performed in accordance with relevant guidelines and regulations.

### Placental tissue collection

The experiments involving human tissue were approved by The Auckland Regional Health and Disability Ethics Committee, New Zealand (NTX/12/06/057/AM03). Preeclamptic placentae (n = 10) and normotensive term placentae (n = 13) were donated by patients at Auckland City Hospital, New Zealand with written consent. Four preeclamptic and four normotensive placentae were used in the deep sequencing experiments and the characteristics of these women from whom these placentae were obtained are shown in Supplementary Table 1. All placentae were delivered from singleton pregnancies. There were no significant differences in maternal age or gestational age at birth between the two groups. Blood pressure (systolic pressure and diastolic pressure), as well as urine protein levels were significantly higher in the preeclampsia group compared to the control group (p < 0.01). Placentae were stored in sterile PBS and transported to the laboratory for immediate processing within 3 hours after either caesarean section or vaginal delivery.

The diagnostic criteria for preeclampsia was new-onset of hypertension after 20 weeks of gestation (defined as systolic blood pressure ≥ 140 mmHg or diastolic blood pressure ≥ 90 mmHg on two occasions, at least 4 hours apart), accompanied with one or more of the following symptoms, new-onset proteinuria (>0.3 gram per 24 hours), new-onset thrombocytopenia (platelet count < 100,000/µl), impaired liver function, renal insufficiency, pulmonary oedema and visual or cerebral disturbances^48^.

### Placental explant culture and trophoblast debris isolation

Explants of approximately 400 mg wet weight were dissected from placentae and cultured in 15 mm diameter Netwell inserts with 440 µm mesh size with Advanced DMEM/F12 medium supplemented with 2% FBS at 37 °C in a humidified atmosphere of air containing 5% CO2 for 17 hours. For small RNA transfection purposes, a mixture of lipofectamine 2000 and 80 nM cy3-labelled siRNA (Thermo Fisher Scientific, AM4621) or miRNA mimic and lipofectamine 2000 (Thermo Fisher Scientific, 4464066) in Opti-MEM® Reduced-Serum Medium (51985042) was added into the placental culture medium. MicroRNA mimic negative control was used as negative experimental control for the transfection. The trophoblast debris was isolated by low speed centrifugation (2000 × g for 5 minutes) of the supernatant collected from the lower chamber. Contaminating red blood cells were removed by incubation in nine volumes of MilliQ water for one minute then the addition of one volume of 10 × PBS to return the trophoblast debris to isotonic conditions. Contaminating CD45+ leukocytes were depleted by using anti-CD45 Dynabeads (Thermo Fisher Scientific, 11153D) according to the manufacturer’s instructions.

### Cell culture

The human microvascular endothelial cell line (HMEC-1) originally derived from dermal microvascular endothelial cells was obtained from the National Centre for Infectious Diseases (USA) and grown in MCDB 131 medium supplemented with 10% FBS, 1% L-glutamine and 1% Penicillin/Streptomycin. The seeding density was 2.5 × 10^4^ cells/cm^2^/mL media. The passages of HMEC-1 used in this study were under 15.

For this work, 1.2 million HMEC-1 cells were exposed to approximately 6600 pieces of trophoblast debris for 21 hours before imaging by confocal microscopy or RNA extraction.

### Visualization of cy3-labelled siRNA

At the end of each experiment, placental explants were removed from the Netwell inserts and embedded in OCT tissue freezing medium contained in tin foil sleeves. The samples were frozen by floating the samples, in metal bottle caps containing isopentane, in liquid nitrogen. The placental tissue sections of 5 µm thickness was cut using a CryoStar™ NX70 Cryostat (Thermo Fisher Scientific) and mounted onto poly-L-lysine coated glass slides, fixed in 4% PFA for 15 minutes and allowed to air dry overnight. Slides of smears of trophoblast debris or coverslips of cultured HMEC-1 cells exposed to cy3-siRNA transfected trophoblast debris were fixed with 4% PFA for 5 minutes.

Placental tissue sections and trophoblast debris smears were counterstained with Hoechst 33342, while HMEC-1 cells were stained with Cell Tracker Green CMFDA (Thermo Fisher Scientific, C34552) and Hoechst nuclear stain. The slides were mounted with anti-fading mounting medium (Electron microscopy science, 17970-25) and images were taken using a confocal microscope (Olympus FluoView™ FV1000).

### Modified total RNA extraction

Total RNA including small RNAs was extracted according to the modified method provided by PureLink mini-kit (Thermo Fisher Scientific, 12183018 A). 1 mL of TRIzol reagent (Thermo Fisher Scientific, 15596026) was used to lyse up to 5 × 10^6^ HMEC-1 cells, the trophoblast debris pellet (collected from 4–6 explants of each individual placenta) or 75 mg of placental tissue. The homogenized samples were incubated at room temperature for 5 minutes before adding 0.2 mL of chloroform. The samples were shaken vigorously for 15 seconds, incubated at room temperature for 3 minutes, and then centrifuged at 12,000 × g at 4 °C for 15 minutes. 500 µL of the upper aqueous phase was transferred to RNase free tubes and mixed with equal volume of 100% ethanol, the mixture was further purified using a PureLink mini-kit according to the manufacturer’s instructions for total RNA extraction. Briefly, samples were vortexed to disperse any precipitate before transfer to a spin cartridge and centrifuged at 12, 000 × g for 1 minute, and the flow-through was discarded. Samples were then washed twice with 500 μL of wash buffer II. RNA was finally eluted using 30 μL of RNase free H_2_O. RNA samples were quantitated by Qubit fluoromentry (Thermo Fisher Scientific) and qualified by TapeStation 4200 (Agilent Genomics).

### Small RNA cDNA library construction and deep sequencing

100 ng of total RNA samples were sent to New Zealand Genomics Limited (NZGL) for small RNA library preparation, using the NEXTflex small RNA sequencing kit V2 (Bioo Scientific) which used a gel cutoff to isolate the small RNA population.

Small RNA sequencing was executed as a service by NZGL on an Illumina HiSeq 2500 System Rapid with 1 × 100 base single end sequencing.

### Small RNA sequence data analysis

The analysis process of the small RNA sequencing data followed a common workflow which included 3′-adapter trimming and QC, sequence alignment, read counting, normalization and expression profiling^49,50^. The data was analysed using a web-based pipeline, iSRAP^51^. Small RNA sequence data was submitted to the GEO repository with reviewer link:

http://www.ncbi.nlm.nih.gov/geo/query/acc.cgi?token = ipcpqiwutpglnif&acc = GSE85926.

### cDNA synthesis and Quantitative RT-PCR

MicroRNA cDNA was reverse transcribed from up to 1 µg of total RNA using a qScript™ microRNA cDNA Synthesis Kit (Quanta Biosciences, 95107-025). The reverse transcription consisted of two reactions: a poly (A) tailing reaction followed by a first strand cDNA synthesis reaction.

MicroRNA cDNA was reverse transcribed from up to 5 µg of RNA using a qScript™ cDNA SuperMix (Quanta Biosciences, 95048-100) according to the manufacturer’s instructions. Reverse transcribed cDNA samples were diluted five times in nuclease free water and stored at −20 °C until use.

The expression of microRNA was quantified by qRT-PCR using PerfeCTa® SYBR® Green FastMix®, Low ROX™ (Quanta Biosciences, 95074-012). The reaction system (10 µL) consisted of 5 µL of PerfeCTa SYBR Green FastMix (2X); 1 µL of PerfeCTa microRNA Assay Primer (2 µM, sequences were included in Supplementary Table 2); 1 µL of PerfeCTa Universal PCR Primer (2 µM); 1 µL of nuclease free water and 2 µL of microRNA cDNA sample. The reaction conditions used a 3-step cycling protocol as follows:

**Pre-incubation/activation**: 95 °C for 2 minutes;

**PCR** (40 cycles)

**Denature**: 95 °C for 5 seconds

**Anneal**: 60 °C for 15 seconds

**Extend**: 70 °C for 15 seconds (collect fluorescence data)

Quantification of the expression of mRNA by qRT-PCR using Platinum SYBR Green qPCR SuperMix UDG w/ROX (Life technologies, 11744500). The reaction system (10 µL) consisted of 1 µL of 2 µM forward primer, 1 µL of 2 µM reverse primer (sequences are included in Supplementary Table 2), 5 µL of SYBR® Green qPCR SuperMix, 3 µL of cDNA in nuclease free water. The reaction conditions were as follows:

**Hold Stage**: 50 °C for 2 minutes, 95 °C for 10 minutes

**PCR Stage**: 40 cycles of 95 °C for 15 seconds, 60 °C for 1 minute.

**Melt Curve Stage**: 95 °C for 15 seconds, 60 °C for 15 seconds and 95 °C for 15 seconds.

The reference genes for the expression of mRNA were *ACTB* and *18 S*. As for miRNA qRT-PCR, due to the unique structure of trophoblast debris, we chose three most invariantly expressed miRNAs (miR-532-5p; miR-92b-3p; miR-29c-5p) across all trophoblast debris samples analysed by sequencing as reference genes (Supplementary Table 5). Quantitative RT-PCR data was analysed by the delta delta Ct method.

### Statistical analysis

Statistical significance of the results was assessed using GraphPad Prism 6 software. Either Mann Whitney test or Wilcoxon matched pairs signed rank test was used as normal distribution cannot be assumed or justified by normality test. Data are presented as median with interquartile range for abnormally distributed data. Statistical significance was considered when P-value < 0.05.

## Electronic supplementary material

Supplementary Tables

Supplementary Figures
