# Supplementary material for: Placental trophoblast debris mediated feto-maternal signalling via small RNA delivery: implications for preeclampsia
Source: Sci Rep. 2017 Oct 31;7:14681. doi: 10.1038/s41598-017-14180-8 (PMC5665858; doi:10.1038/s41598-017-14180-8)
Supplement: Supplementary file 1 — Supplementary Tables [file 41598_2017_14180_MOESM1_ESM.doc]

Placental trophoblast debris mediated feto-maternal signalling via small RNA delivery: implications for preeclampsia

Jia Wei^1^*, Cherie Blenkiron^2.3^, Peter Tsai^3^, Joanna L. James^1^, Qi Chen^1^, Peter R. Stone^1^, Lawrence W. Chamley^1^

1. Department of Obstetrics and Gynaecology, The University of Auckland, New Zealand;
2. Department of Surgery, The University of Auckland, New Zealand;
3. Department of Molecular Medicine and Pathology, The University of Auckland, New Zealand

Corresponding author:

Dr. J Wei

Email: [j.wei@auckland.ac.nz](mailto:j.wei@auckland.ac.nz)

**Supplementary Table 1: Characteristics of the study cohort for the small RNA sequencing project**

|  | **Preeclampsia (n=4)** | **Control (n=4)** |
| --- | --- | --- |
| **Maternal age (years)** | 28.0 ± 5.78 | 32 ± 4.99 |
| **Nulliparous** | 4/4 (100%) | 2/4 (50%) |
| **Gestational age at birth (weeks)** | 32.65 ± 2.94 | 36.65 ± 1.60 |
| **Systolic blood pressure (mm Hg)** | 174 ± 11.12 | 108 ± 14.07 ^*^ |
| **Diastolic blood pressure (mm Hg)** | 107.5 ± 5.72 | 75.5 ± 4.92 ^*^ |
| **Proteinuria( > 0.3 g/day)** | 4/4 (100%) | 0/4 (0%) ^*^ |
| **PE onset (gestational week)** | 31.4 ± 1.59 | N/A |

*- P_Mann_ <0.01

**Supplementary Table 2: Primer sequences for miRNA qRT-PCR and mRNA qRT-PCR**

| **hsa-miR-497-5p** | | CAGCAGCACACUGUGGUUUGU |
| --- | --- | --- |
| **hsa-miR-615-3p** | | UCCGAGCCUGGGUCUCCCUCUU |
| **hsa-miR-455-5p** | | UAUGUGCCUUUGGACUACAUCG |
| **hsa-miR-526b-5p** | | CUCUUGAGGGAAGCACUUUCUGU |
| **hsa-miR-145-5p** | | GUCCAGUUUUCCCAGGAAUCCCU |
| **hsa-miR-1247-5p** | | ACCCGUCCCGUUCGUCCCCGGA |
| **hsa-miR-532-5p** | | CAUGCCUUGAGUGUAGGACCGU |
| **hsa-miR-92b-3p** | | UAUUGCACUCGUCCCGGCCUCC |
| **hsa-miR-29c-5p** | | UGACCGAUUUCUCCUGGUGUUC |
| ***BIRC3*** | Forward | 5’-CTGGAAAAGAGGAGACAGTCCT-3’ |
|  | Reverse | 5’-ACTGTTTTCTGTACCCGGAAGT-3’ |
| ***ANGTP2*** | Forward | 5’-GAACCAGACGGCTGTGATGA-3’ |
|  | Reverse | 5’-TTAATACTTGGGCTTCCACATCAG-3’ |
| ***MMP9*** | Forward | 5’-CATTCAGGGAGACGCCCATT-3’ |
|  | Reverse | 5’-AACCGAGTTGGAACCACGAC-3’ |
| ***ACTB*** | Forward | 5’-GCGGACTATGACTTAGTTGCGTTA-3’ |
|  | Reverse | 5’-CATCTTGTTTTCTGCGCAAGTT-3’ |
| ***18S*** | Forward | 5’-CTACCACATCCAAGGAAGCA-3’ |
|  | Reverse | 5’-TTTTTCGTCACTACCTCCCCG-3’ |

**Supplementary Table 3: Differential expression of rRNA fragments** between preeclamptic and normotensive trophoblast debris using iSRAP

| **Chromosome** | **Genomic position (Start)** | **Genomic position (End)** | **ID** | **Log2 fold change** | **Adjusted p-value** |
| --- | --- | --- | --- | --- | --- |
| chr14 | 75352884 | 75352924 | 5S | -3.051 | 0.001 |
| chr8 | 28530410 | 28530499 | 5S | -2.883 | 0.001 |
| chr13 | 106155400 | 106155490 | 5S | -2.879 | 0.001 |
| chr22 | 32635109 | 32635198 | 5S | -2.845 | 0.001 |
| chrUn_KI270442v1 | 380607 | 380728 | 5S | -2.914 | 0.001 |
| chr8 | 8831418 | 8831487 | 5S | -2.768 | 0.001 |
| chr6 | 106449378 | 106449463 | 5S | -2.791 | 0.001 |
| chr16 | 9564408 | 9564497 | 5S | -2.727 | 0.001 |
| chr6 | 116178246 | 116178333 | 5S | -2.806 | 0.001 |
| chr3 | 63545439 | 63545520 | 5S | -2.703 | 0.001 |
| chr12 | 133047540 | 133047625 | 5S | -2.667 | 0.001 |
| chr8 | 138803379 | 138803450 | 5S | -2.632 | 0.001 |
| chr17 | 10886298 | 10886383 | 5S | -2.652 | 0.001 |
| chr8 | 17748117 | 17748208 | 5S | -2.721 | 0.001 |
| chr6 | 153420440 | 153420526 | 5S | -2.584 | 0.001 |
| chr5 | 33277860 | 33277953 | 5S | -2.574 | 0.002 |
| chr17 | 66267742 | 66267831 | 5S | -2.732 | 0.002 |
| chr11 | 21000880 | 21000965 | 5S | -2.478 | 0.002 |
| chr12 | 38161463 | 38161582 | 5S | -2.237 | 0.002 |
| chr4 | 190015137 | 190015257 | 5S | -2.804 | 0.002 |
| chr6 | 4427962 | 4428082 | 5S | -2.176 | 0.002 |
| chr8 | 90511135 | 90511241 | 5S | -2.453 | 0.002 |
| chr17 | 15782341 | 15782415 | 5S | -3.108 | 0.002 |
| chr19 | 21113125 | 21113235 | 5S | -2.219 | 0.003 |
| chr1 | 228619229 | 228619350 | 5S | -2.216 | 0.003 |
| chr10 | 106970021 | 106970110 | 5S | -2.272 | 0.003 |
| chr1 | 228632628 | 228632749 | 5S | -2.205 | 0.003 |
| chr3 | 151188098 | 151188184 | 5S | -2.230 | 0.003 |
| chr4 | 352668 | 352788 | 5S | -2.591 | 0.003 |
| chr1 | 228634868 | 228634989 | 5S | -2.188 | 0.003 |
| chr19 | 57867262 | 57867352 | 5S | -2.237 | 0.003 |
| chr1 | 228612506 | 228612627 | 5S | -2.166 | 0.003 |
| chr14 | 103316946 | 103317036 | 5S | -2.332 | 0.003 |
| chr1 | 228610265 | 228610386 | 5S | -2.175 | 0.003 |
| chr1 | 228614747 | 228614868 | 5S | -2.164 | 0.003 |
| chr9 | 112252257 | 112252347 | 5S | -2.477 | 0.003 |
| chr1 | 228616988 | 228617109 | 5S | -2.168 | 0.003 |
| chr4 | 91264681 | 91264769 | 5S | -2.610 | 0.003 |
| chr1 | 228630387 | 228630508 | 5S | -2.150 | 0.003 |
| chr3 | 181822870 | 181822990 | 5S | -2.139 | 0.003 |
| chr1 | 228621444 | 228621565 | 5S | -2.152 | 0.003 |
| chr1 | 228646037 | 228646158 | 5S | -2.151 | 0.003 |
| chr1 | 228625906 | 228626027 | 5S | -2.147 | 0.003 |
| chr1 | 228637093 | 228637214 | 5S | -2.123 | 0.003 |
| chr1 | 228641565 | 228641686 | 5S | -2.134 | 0.003 |
| chr1 | 228623664 | 228623785 | 5S | -2.126 | 0.003 |
| chr1 | 228643806 | 228643927 | 5S | -2.114 | 0.003 |
| chr1 | 228639334 | 228639455 | 5S | -2.100 | 0.003 |
| chr1 | 78375188 | 78375267 | 5S | -2.332 | 0.003 |
| chr18 | 24170502 | 24170623 | 5S | -2.141 | 0.004 |
| chr14 | 271683 | 271767 | 5S | -2.227 | 0.006 |
| chr1 | 228558283 | 228558364 | 5S | -1.984 | 0.007 |
| chr2 | 20401636 | 20401724 | 5S | -2.349 | 0.007 |
| chr12 | 118829680 | 118829770 | 5S | -2.383 | 0.007 |
| chr8 | 59455884 | 59455974 | 5S | -2.167 | 0.007 |
| chr14 | 24158602 | 24158724 | 5S | -2.195 | 0.007 |
| chr1 | 64689119 | 64689158 | 5S | -2.242 | 0.007 |
| chr18 | 9844759 | 9844829 | 5S | -2.302 | 0.007 |
| chr16 | 66301808 | 66301898 | 5S | -2.539 | 0.007 |
| chr10 | 14663404 | 14663494 | 5S | -2.093 | 0.010 |
| chrY | 10092872 | 10092993 | 5S | -1.977 | 0.012 |
| chr14 | 91803888 | 91803972 | 5S | -2.044 | 0.013 |
| chr9 | 72299752 | 72299833 | 5S | -2.635 | 0.013 |
| chr1 | 87453283 | 87453373 | 5S | -2.273 | 0.013 |
| chrX | 69672478 | 69672599 | 5S | -1.677 | 0.015 |
| chr14 | 75604172 | 75604262 | 5S | -2.318 | 0.015 |
| chr2 | 11561664 | 11561779 | 5S | -2.104 | 0.015 |
| chr1 | 169067263 | 169067340 | 5S | -2.732 | 0.016 |
| chr2 | 142680431 | 142680473 | 5S | -2.536 | 0.019 |
| chr4 | 56097390 | 56097479 | 5S | -2.055 | 0.023 |
| chr16 | 84262836 | 84262928 | 5S | -2.673 | 0.026 |
| chr9 | 99145876 | 99145965 | 5S | -1.855 | 0.028 |
| chr1 | 39207761 | 39207803 | 5S | -2.484 | 0.029 |
| chr6 | 120686620 | 120686712 | 5S | -1.751 | 0.030 |
| chr1 | 228628145 | 228628266 | 5S | -1.562 | 0.038 |
| chr13 | 36864381 | 36864456 | 5S | -1.783 | 0.039 |
| chr7 | 36593683 | 36593769 | 5S | -2.418 | 0.040 |
| chr13 | 21369810 | 21369897 | 5S | -1.802 | 0.040 |
| chr12 | 99083815 | 99083904 | 5S | -2.017 | 0.046 |
| chr14 | 77403904 | 77403993 | 5S | -2.036 | 0.046 |
| chr12 | 13440909 | 13441001 | 5S | -1.796 | 0.048 |
| chr9 | 19492827 | 19492990 | LSU-rRNA_Hsa | 3.036 | 0.003 |
| chrM | 2589 | 2745 | LSU-rRNA_Hsa | 1.742 | 0.032 |
| chr18 | 80012846 | 80013065 | LSU-rRNA_Hsa | 1.759 | 0.035 |
| chr19 | 22694812 | 22694856 | SSU-rRNA_Hsa | 2.443 | 0.040 |

**Supplementary Table 4 Fragments of tRNA and snRNA that were present in significantly different levels between preeclamptic and normotensive trophoblast debris.**

| **Chromosome** | **Genomic position (Start)** | **Genomic position**  **(End)** | **ID** | **DESeq2** | | **edgeR** | | **Voom** | |
| --- | --- | --- | --- | --- | --- | --- | --- | --- | --- |
|  |  |  |  | **Log2 FC *** | **Adj P-value †** | **Log2 FC *** | **FDR ‡** | **Log2 FC *** | **Adj P-value †** |
| chr6 | 28607155 | 28607227 | tRNA-Ala-AGC | -1.95 | 0.003 | -2.30 | 0.007 | -1.99 | 0.042 |
| chr6 | 28710588 | 28710660 | tRNA-Ala-AGC | -2.02 | 0.002 | -2.35 | 0.004 | -2.07 | 0.03 |
| chr6 | 28719703 | 28719775 | tRNA-Ala-AGC | -2.05 | 0.002 | -2.41 | 0.005 | -2.17 | 0.028 |
| chr13 | 41455924 | 41455996 | tRNA-Glu-CTC | 2.59 | <0.001 | 2.88 | 0.002 | 2.38 | 0.037 |
| chr6 | 28478622 | 28478704 | tRNA-Leu-AAG | -1.73 | 0.006 | -1.96 | 0.011 | -1.87 | 0.03 |
| chr8 | 135742364 | 135742495 | U1 | 2.58 | 0.027 | 3.85 | 0.008 | 2.77 | 0.007 |
| chr1 | 145281115 | 145281281 | U1 | 2.12 | 0.016 | 2.44 | 0.025 | 2.65 | 0.03 |
| chr12 | 62850738 | 62850898 | U1 | 2.12 | 0.001 | 2.29 | 0.008 | 2.02 | 0.046 |
| chr11 | 65147588 | 65147774 | U2 | 2.16 | 0.016 | 2.45 | 0.025 | 2.62 | 0.028 |
| chr1 | 92700867 | 92700934 | U4 | 3.35 | 0.002 | 6.07 | 0.001 | 3.55 | 0.001 |
| chr2 | 50064641 | 50064700 | U5 | 3.41 | 0.002 | 6.09 | 0.001 | 3.21 | 0.002 |
| chr11 | 12787924 | 12787962 | U5 | 3.4 | 0.001 | 4.89 | 0.003 | 3.31 | 0.003 |
| chr1 | 11909806 | 11909926 | U5 | 2.45 | 0.022 | 3.19 | 0.018 | 2.90 | 0.011 |
| chr4 | 55028861 | 55028914 | U5 | 2.55 | 0.043 | 5.21 | 0.012 | 2.57 | 0.016 |
| chr8 | 130584847 | 130584894 | U5 | 2.36 | 0.031 | 3.08 | 0.037 | 2.57 | 0.039 |
| chr5 | 109014833 | 109014940 | U6 | 2.63 | 0.011 | 3.43 | 0.011 | 3.04 | 0.007 |
| chr11 | 200830008 | 200830116 | U8 | 3.78 | <0.001 | 4.36 | <0.001 | 4.32 | 0.001 |
| chr1 | 123301391 | 123301524 | U8 | 2.25 | 0.002 | 2.43 | 0.006 | 2.23 | 0.046 |
|  |  |  |  |  |  |  |  |  |  |

**Log2 FC***: Log2 fold change

**Adj P-value †:** Adjusted p-value

**FDR ‡**: False discovery rate

**Supplementary Table 5: Reads per million for three miRNAs among either preeclamptic or normotensive trophoblast debris**

|  | **PE TD 1** | **PE TD 2** | **PE TD 3** | **PE TD 4** | **Normotensive**  **TD 1** | **Normotensive**  **TD 2** | **Normotensive**  **TD 3** | **Normotensive**  **TD 4** |
| --- | --- | --- | --- | --- | --- | --- | --- | --- |
| hsa-miR-532-5p | 441.74 | 497.36 | 406.79 | 462.20 | 434.97 | 418.33 | 454.54 | 502.89 |
| hsa-miR-92b-3p | 132.93 | 126.20 | 145.56 | 136.80 | 94.99 | 128.76 | 112.59 | 193.47 |
| hsa-miR-29c-5p | 14.94 | 10.10 | 14.18 | 12.09 | 12.72 | 9.62 | 19.95 | 10.56 |

**PE:** Preeclamptic

**TD:** trophoblast debris
