# Supplementary material for: Placental trophoblast debris mediated feto-maternal signalling via small RNA delivery: implications for preeclampsia
Source: Sci Rep. 2017 Oct 31;7:14681. doi: 10.1038/s41598-017-14180-8 (PMC5665858; doi:10.1038/s41598-017-14180-8)

Placental trophoblast debris mediated feto-maternal signalling via small RNA delivery: implications for preeclampsia

Jia Wei[1]*, Cherie Blenkiron[2,3], Peter Tsai[3], Joanna L. James[1], Qi Chen[1], Peter R. Stone[1], Lawrence W. Chamley[1]

1. Department of Obstetrics and Gynaecology, The University of Auckland, New Zealand;
2. Department of Surgery, The University of Auckland, New Zealand;
3. Department of Molecular Medicine and Pathology, The University of Auckland, New Zealand

Corresponding author:

Dr. J Wei

Email: j.wei@auckland.ac.nz

Supplementary figure legend:

Supplementary figure 1: RNA electrophoresis of RNA samples isolated from trophoblast debris and the placental explants from which the debris samples were derived

Total RNA samples were isolated from four individual trophoblast debris (TD) and the matched placental tissue lysate (P) from which the debris was derived. These RNA samples were assessed using Agilent high sensitivity and normal sensitivity RNA ScreenTape assay. Gel image (A) shows RNA electrophoresis of the RNA isolated from four preclamptic trophoblsat debris and the placental explants from which the debris was derived. Representative electropherogram of RNA sample from preeclamptic trophoblast debris (B) and the matched donor placental explants (C) shows the RNA length distribution with 18/28S peaks annotated.

Supplementary figure 2: Visualizing delivery of fluorescent siRNA to endothelial cells by trophoblast debris

HMEC-1 cells were exposed to untreated trophoblast debris (upper lane) or cy3-siRNA (red) transfected trophoblast debris (lower lane) for 21 hours before co-staining with Cell Tracker Green (CMFDA, green) and DAPI (blue). Scale bars represent 30 μm in all images.

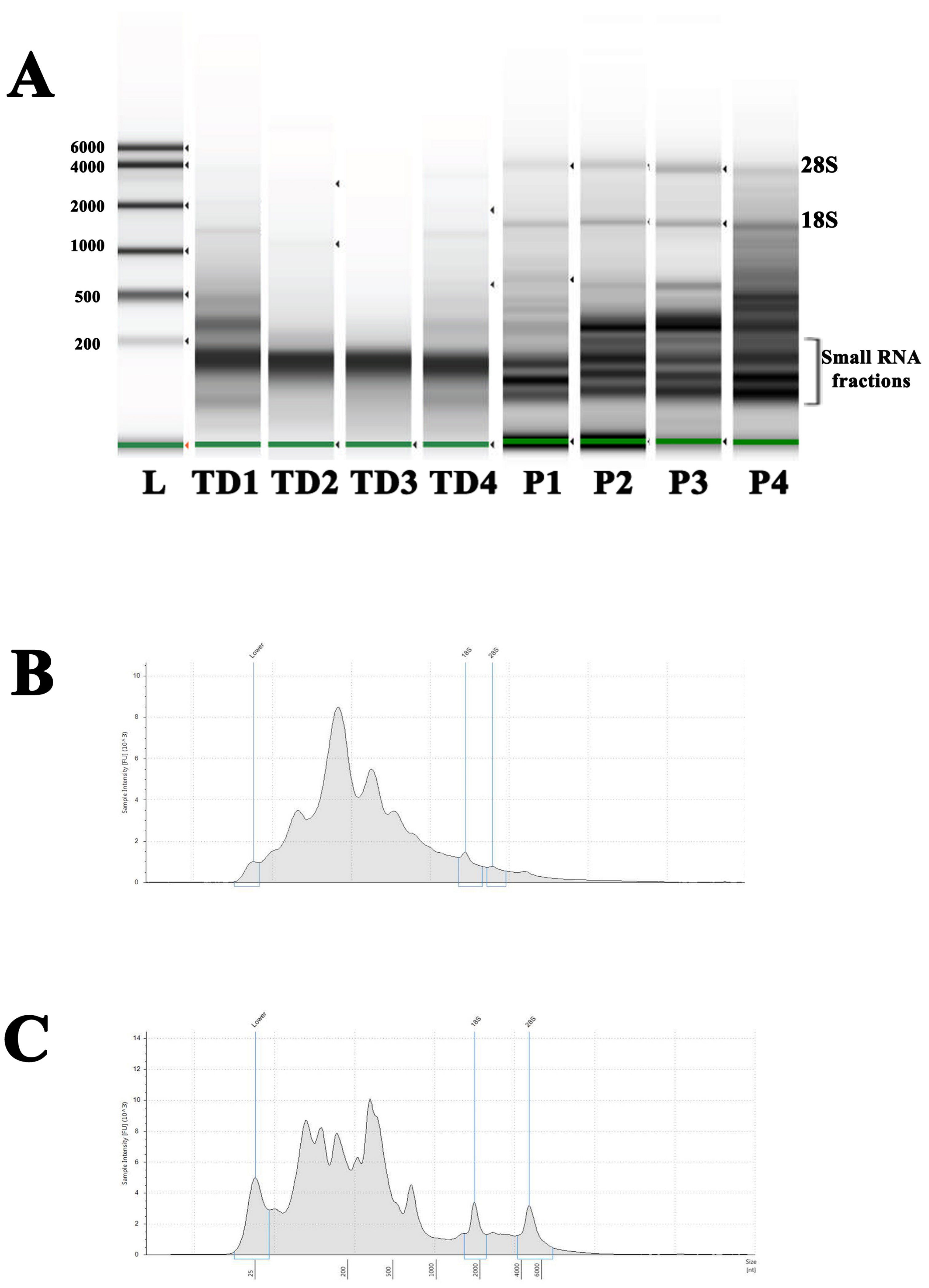

1

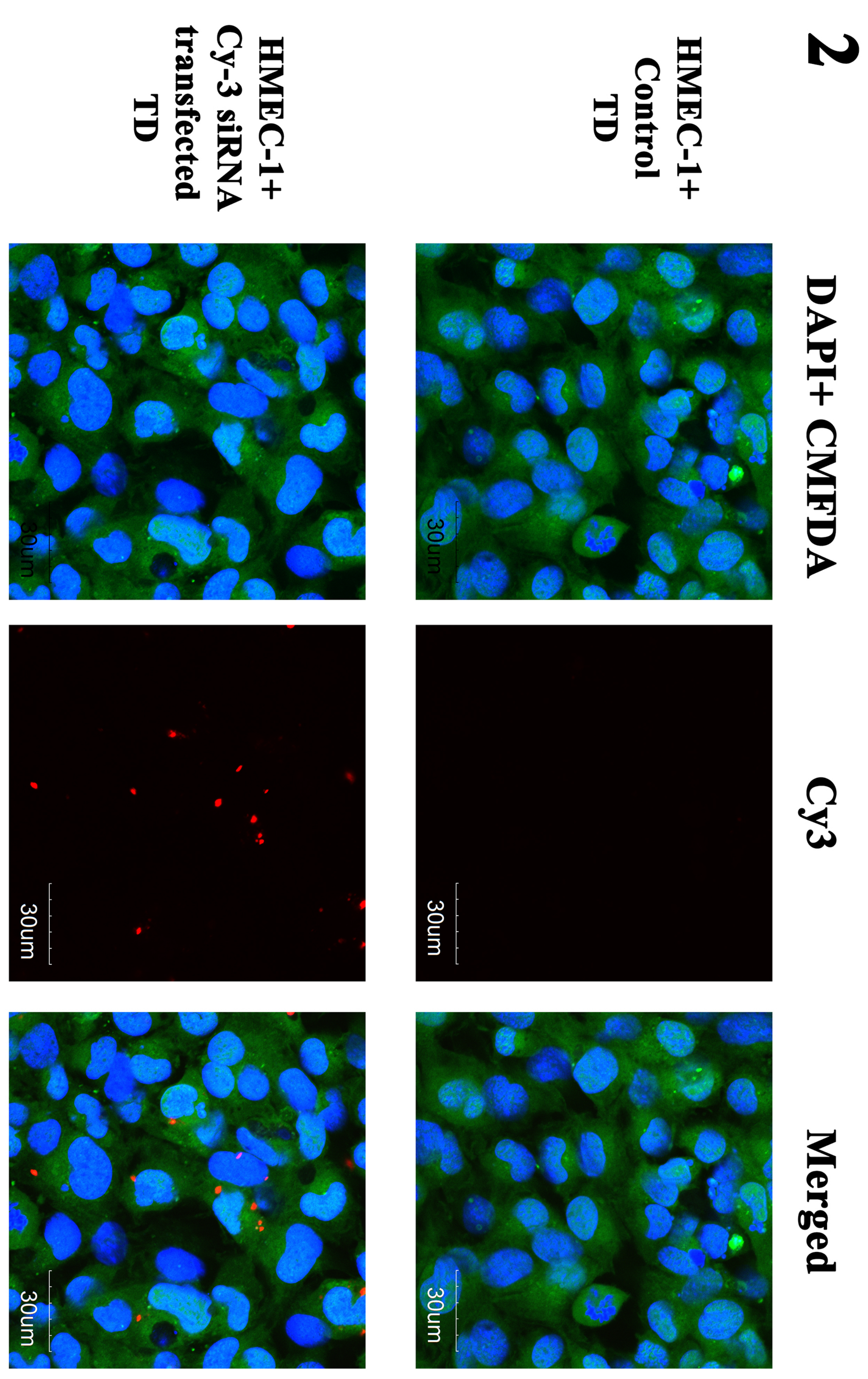

Supplement: Supplementary file 2 — Supplementary Figures [file 41598_2017_14180_MOESM2_ESM.pdf]
